# *In Vitro* Permeation of Micronized and Nanonized Alaptide from Semisolid Formulations

**DOI:** 10.1155/2013/787283

**Published:** 2013-12-18

**Authors:** Radka Opatrilova, Aneta Cernikova, Lenka Coufalova, Jiri Dohnal, Josef Jampilek

**Affiliations:** Department of Chemical Drugs, Faculty of Pharmacy, University of Veterinary and Pharmaceutical Sciences, Palackeho 1/3, 61242 Brno, Czech Republic

## Abstract

This study is focused on *in vitro* permeation of the original Czech compound, a skin/mucosa tissue regeneration promoter, known under the international nonproprietary name “alaptide,” in micronized and nanonized forms. Alaptide showed a great potential for local applications for treatment and/or regeneration of the injured skin. The above mentioned technological modifications influence the permeation of alaptide through artificial or biological membranes, such as PAMPA or skin. The permeation of micronized and nanonized form of alaptide formulated to various semisolid pharmaceutical compositions through full-thickness pig ear skin using a Franz cell has been investigated in detail. In general, it can be concluded that the nanonized alaptide permeated through the skin less than the micronized form; different observations were made for permeation through the PAMPA system, where the micronized form showed lower permeation than the nanonized alaptide.

## 1. Introduction

**Figure 1:**
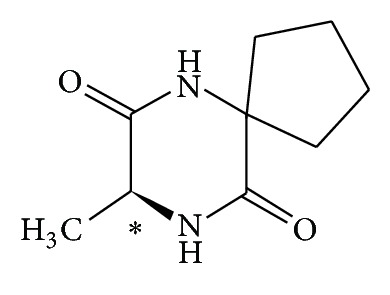
Structure of (*S*)-alaptide.

It is known that damage or deficit of skin and mucosa, such as injury, wound, morsus, scald, burn, congelation, radiation injury, ultraviolet irradiation, electric injury, traumatic injury, skin ulcer, bedsore, and bullous skin diseases, causes degenerative exfoliation, necrosis, apoptosis, or apoptosis-like cell death of skin tissue-composing cells or mucosal tissue-composing cells. As for effective methods for prevention or therapy of diseases caused by mechanical or physical damage or defect of the skin tissue or the mucosal tissues, application of drugs which can rapidly regenerate and/or reconstruct the degenerated and defected skin tissues, mucosal tissues, and composing cells thereof and promote wound healing is considered [1–3].

(*S*)-8-Methyl-6,9-diazaspiro[4.5]decan-7,10-dione, known under the international nonproprietary name (INN) “alaptide” (see Figure 1), can be also considered as skin/mucosa tissue regeneration promoters. It was designed as an analogue of melanocyte-stimulating hormone release-inhibiting factor (MIF) and synthesized by Kasafirek et al. at the Research Institute for Pharmacy and Biochemistry in the 80s of the 20th century [4–7]. Alaptide is a white crystalline compound, generally poorly soluble, stable in the sunlight, and storable at ambient temperature [8, 9]. Since it is an optically active molecule, it is necessary to inspect the entire synthetic process with the intention to determine unambiguously the absolute configuration of the final product. A liquid chromatographic method for control of alaptide enantiopurity was developed [10]. It was found out that when optically pure starting compounds are used, no change of configuration (racemization) occurs, and the chirally pure final product is obtained. Except for the recently published X-ray powder diffraction data revealing the unit cell parameters and the space group of alaptide crystals [11], absolute configuration of the molecule was also determined by electronic circular dichroism spectroscopy [12].

The influence of alaptide on epidermal regeneration was investigated in a number of tests. *In vivo* experiments were performed using pigs, to which alaptide was applied on experimental injury, and faster skin regeneration was observed after the application of alaptide. Similarly, alaptide accelerated curing experimental skin injuries on rats [8]. Alaptide probably negatively affects the inhibition of release of melanocyte-stimulating hormone, and thus it increases concentration of melanocytes in epidermis. Melanocytes significantly influence creation and function of keratinocytes by means of melanosomas [13, 14]. Keratinocytes migrate from *stratum basale* to *stratum spinosum* and *stratum granulosum* to *stratum corneum*, where they support epidermis regeneration [15]. It is excreted unchanged, mostly via urine; a similar metabolic profile was also found in man [8, 16, 17]. No toxic effects of alaptide (including skin irritation) were observed. Induction of biotransformation enzymes CYP1A1, CYP1A2, and CYP1B1 in hepatocytes did not occur [8, 9, 16, 18].

Based on the above mentioned facts, it can be summarized that alaptide showed a great potential for local applications for treatment and/or regeneration of injured skin or mucosa [8, 19]. The aim of this paper is to evaluate the rate of permeation of alaptide through biological membranes. As penetration/permeation of compounds to/through the membranes is important to know especially at local applications and micro- and nanonized substances feature different behavior related to penetration/permeation [20–24], micronized and nanonized forms of (*S*)-alaptide were prepared, and permeation of both forms of alaptide through the membrane was also evaluated using the PAMPA (parallel artificial membrane permeability assay) technique [25–27]. With respect to external application of both forms, *in vitro* ability to permeate through full-thickness pig ear skin was also evaluated using a Franz cell [28, 29], including permeation from various semisolid formulations.

## 2. Material and Methods

### 2.1. Preparation of Micronized Alaptide

Alaptide was synthesized by the standard process, as described above [4, 5, 8]. Then it was micronized. The particle size of the used micronized alaptide was measured by a microscope NIKON Optiphot 2 with a digital camera VDS CCD-1300F. The particle size distribution (x_90_) of microcrystalline alaptide was 40 *μ*m.

### 2.2. Preparation of Nanonized Alaptide

The suspension of micronized alaptide (30 g), polyvinylpyrrolidone (30 g), and purified water (240 mL, during milling was diluted by addition of additional 150 mL) was initially mixed for 12 h at ambient temperature and then filtered through a mill sieve. The milling procedure was performed using a nanomill NETZSCH (Germany) with glass beads (0.3 mm); the rotor speed was 986 rpm; the pump speed was 30 rpm; the temperature in the grinding chamber was within 17–20°C. The rotor speed was increased to 1500 rpm after 6 h of milling. The total time of milling was 57.5 h. The content of alaptide in the suspension was 38.76 g/L (determined by RP-HPLC; see below). The particle size of the prepared nanonized alaptide was measured by Sympatec NANOPHOX 0138 P (Germany), and the particle size x_90_ was 770 nm.

### 2.3. Investigated Semisolid Formulations

Directly before the application, nanonized alaptide was dispersed by ultrasonics to formulations to avoid possible reagglomeration. The composition of gel with alaptide 1% (w/w) was the following: alaptide 1 g, carboxymethylcellulose ointment (carboxymethylcellulose sodium 5 g, Macrogol 300 10 g, propylene glycol 2.5 g, methylparaben 0.2 g, propylparaben 0.2 g, and purified water 87.3 g) up to 100 g. The composition of cream with alaptide 1% (w/w) was the following: alaptide 1 g, cremor neoaquasorb 95 g, and propylene glycol up to 100 g. The composition of ointment with alaptide 1% (w/w) was as follows: alaptide 1 g, cera lanae hydrosa 75 g, yellow vaseline 20 g, and liquid paraffin up to 100 g.

### 2.4. Biology

#### 2.4.1. *In Vitro* PAMPA Experiments

The permeability of micronized and nanonized alaptide was evaluated *in vitro*, using a vertical PAMPA (parallel artificial membrane permeability assay) system (BD gentest precoated PAMPA plate system, 96 wells). The PAMPA system constitutes a lipophilic membrane, which surface is coated by phospholipids that simulate intestinal wall. Micronized alaptide (10 mg) or the amount of nanosuspension corresponding to 10 mg of alaptide was weighed. The donor samples were prepared by dissolving the tested samples in 40 mL of 0.01 M HCl and after 15 min pH was adjusted to pH 6 using bicarbonate buffer. A carbonate buffer saline (physiological solution) with pH 7.4 was used as a receptor phase. About 0.5 h before the experiment, the PAMPA system was taken out from the freezer and warmed up to the ambient temperature. The receptor phase (200 *μ*L/well) was pipetted into the upper wells. The donor phase was pipetted into the lower wells (300 *μ*L/well). After the incubation time (5 h) 10 *μ*L of the acceptor phase was taken from each well and mixed with physiological solution (990 *μ*L). At least five determinations were performed.

Analysis of samples for alaptide content was performed using an Agilent 1200 series HPLC system, equipped with a diode array detection (DAD) system, a quaternary model pump, and an automatic injector (Agilent Technologies, Germany). Data acquisition was performed using ChemStation chromatography software. A Waters Symmetry C_8_ 5 *μ*m, 4.6 × 250 mm (Waters Corp., Milford, MA, USA) chromatographic column was used. A mixture of MeCN (HPLC grade, 50.0%) and H_2_O (HPLC—Mili-Q Grade, 50.0%) was used as a mobile phase. The total flow of the column was 0.5 mL/min, injection 10 *μ*L, column temperature 24°C, and sample temperature 10°C. The detection wavelength of 204 nm was chosen; the time of analysis was 10 min. The retention time (*t*_*R*_) of alaptide was 3.1 ± 0.05 min, the limit of detection (LOD) was 6.8 ng/mL, and the limit of quantification (LOQ) was 23.0 ng/mL.

#### 2.4.2. *In Vitro* Transdermal Permeation Experiments Performed Using Franz Diffusion Cell

Skin samples were obtained from porcine ear. Full-thickness dorsal skin was cut in fragments and stored at −20°C until utilized. Skin samples were slowly thawed (at 4°C overnight and then at ambient temperature) prior to each experiment. The permeation through the skin of the micronized alaptide alone (1 mL of suspension with the alaptide concentration of 1%), nanonized alaptide alone (in the amount corresponding to 1% concentration of micronized alaptide), and both forms of alaptide incorporated into ointment, cream, or gel was evaluated *in vitro*, using a vertical Franz diffusion cell (SES-Analytical Systems, GmbH, Germany), with a donor surface area of 63.585 mm^2^ and a receptor volume of 5.2 mL. The skin was mounted between the donor and receptor compartments of the Franz diffusion cell with the epidermal side up. To the donor part of the Franz diffusion cell with the volume of 1 mL an investigated sample was applied in the form of solution, suspension, emulsion, gel, cream, or ointment, and then the donor compartment of the cell was covered by Parafilm. The receptor compartment was filled with phosphate buffered saline (pH 7.4) and was maintained at 34 ± 0.5°C, while using circulating water bath. The receptor compartment content was continuously stirred, using a magnetic stirring bar. The skin was kept in contact with the receptor phase for 0.5 h at 34 ± 0.5°C prior to the experiment. Samples (0.5 mL) of the receptor phase were withdrawn at six predetermined time intervals over 24 h (0.5, 1, 2, 4, 6, and 24 h), and the cell was refilled with an equivalent amount of fresh buffer solution. A minimum of five determinations were performed using skin fragments from a minimum of 2 animals for each compound. The samples were immediately analyzed by the HPLC method using the same conditions as described previously. The results of alaptide permeation are summarized in Table 1.

#### 2.4.3. Statistical Evaluation

All experiments were carried out 5-fold. Data were expressed as means ± SD. Differences were evaluated by one-way analysis of the variance (ANOVA) test completed by Bonferroni's multicomparison test (ORIGIN PRO7). The differences were considered significant at *P* = 0.05. The independent variables and responses (flux and lag time) of all model samples were analyzed using ORIGIN PRO7.

## 3. Results and Discussion

### 3.1. *In Vitro* PAMPA Experiments

The preliminary permeability screening of micronized and nanonized alaptide, which was obtained by milling process with glass beads, was performed using polyvinylidene fluoride (PVDF), that is, using PAMPA that has become a very useful and quite cheap tool for predicting intestinal permeability and is well suited as a ranking tool for the assessment of the compounds with passive intestinal transport mechanisms [25–27]. The permeability of nanoalaptide through PAMPA after 5 h (0.6 mg ± 0.01) was 2-fold higher than that of micronized alaptide (0.3 mg ± 0.01). Both values of permeability are expressed as mean ± SD (*n* = 5 experiments).

The results of PAMPA experiments and testing in Franz cells (see below) differed completely. It seems that the permeation of micronized and nanonized alaptide is most significantly influenced by the use of the real skin as a barrier in Franz cells and artificial PVDF membrane in PAMPA. The major drawback of the PAMPA technique is that it can only predict passive diffusion and is therefore unable to generate the full description of the permeability process at the real skin. The skin is a complex organ that can influence and change pharmacokinetics of the administered drugs by a number of various interactions [29, 30]. On the other hand, PAMPA is an excellent tool to rapidly predict passive permeability through the gastrointestinal tract with high throughput efficiency, as mentioned above. Other parameters that may influence the results are solubility, supramolecular superassembly properties of nanonized alaptide, and nanoparticle stabilizer properties, but the explanation of these parameters is not the aim of this study.

### 3.2. *In Vitro* Transdermal Permeation Experiments Performed Using Franz Diffusion Cell

The main focus was the investigation of nanonized and micronized alaptide permeation through porcine ear skin. Firstly, both forms were evaluated using a phosphate buffer (pH 7.4) as a control donor inert vehicle that did not support permeation; consequently, both forms were incorporated into semisolid formulations. Pharmaceutical compositions were prepared in the form of ointment, cream, and gel with the content of alaptide in concentration 1% (w/w). Alaptide was used in microcrystalline form or in the form of nanoparticles. Porcine ear skin was selected for initial evaluation of permeation, as this tissue is a suitable *in vitro* model of human skin [31, 32]. Porcine skin proved to be histologically and biochemically similar to human skin; therefore, full-thickness pig ear skin has been used in numerous percutaneous absorption studies [33]. The skin permeation experiments were performed using static Franz diffusion cells [28]. As it could be expected, the presented transdermal permeation results of micronized and nanonized alaptide and the data obtained using PAMPA method are different. The principal difference is due to different properties of the transport “membrane;” the PAMPA diaphragm is an artificial model of the cell lipid bilayer “simulating” the intestinal wall, whereas the ear porcine skin is a much more complex naturally constructed barrier, as discussed above.

In transdermal experiments, as a result of the sampling of large volumes from the receptor medium (and replenishment with equal volumes of the fresh medium), the receptor medium was constantly being diluted. Consequently, the receptor compartment concentration of alaptide was corrected for sample removal and replenishment using the following:

(1) $$\begin{array}{c} C_{n}^{\prime }=C_{n}\left(\frac{V_{t}}{V_{t}-V_{s}}\right)\left(\frac{C_{n-1}^{\prime }}{C_{n-1}}\right), \end{array}$$

where *C*_*n*_′ is corrected drug concentration in the *n*th sample, *C*_*n*_ is measured drug concentration in the *n*th sample, *C*_*n*−1_′ is corrected drug concentration in the (*n* − 1)th sample, *C*_*n*−1_ is measured drug concentration in the (*n* − 1)th sample, *V*_*t*_ is total volume of receptor solution, *V*_*s*_ is volume of the sample, and *C*_1_′ is *C*_1_ [34].

The corrected data were expressed as the cumulative drug permeation (*Q*_*t*_) per unit of skin surface area using the following:

(2) $$\begin{array}{c} Q_{t}=\frac{C_{n}^{\prime }}{A}, \end{array}$$

where *A* = 0.6359 cm^2^ in our experiment.

If the cumulative amount of drug per unit area (*Q*_*t*_ [*μ*g]) in the receiver chamber is plotted as a function of time (*T* [h]), steady state permeation flux (*J* [*μ*g/h/cm^2^]) can be calculated from the slope of the linear portion of the curve [35]. It takes some time for achieving the steady state of maximum permeation and this time may be considered to be the lag time (*T*_lag_ [h]). The lag time can be determined by extrapolating the linear portion of cumulative amount of permeation per unit area (*Q*_*t*_) versus time (*T* [h]) curve to the abscissa [36].

Permeability coefficient (*K*_*p*_ [cm/h]) can be calculated according to the following:

(3) $$\begin{array}{c} K_{p}=\frac{\;J}{C_{d}}, \end{array}$$

where *C*_*d*_ is drug concentration in the donor compartment (in our experiment 1.0% w/w, i.e., 1.0 × 10^4^ *μ*g/mL). It is assumed that, under sink conditions, drug concentration in the receptor compartment is negligible compared to that in the donor compartment [37, 38].

**Figure 2:**
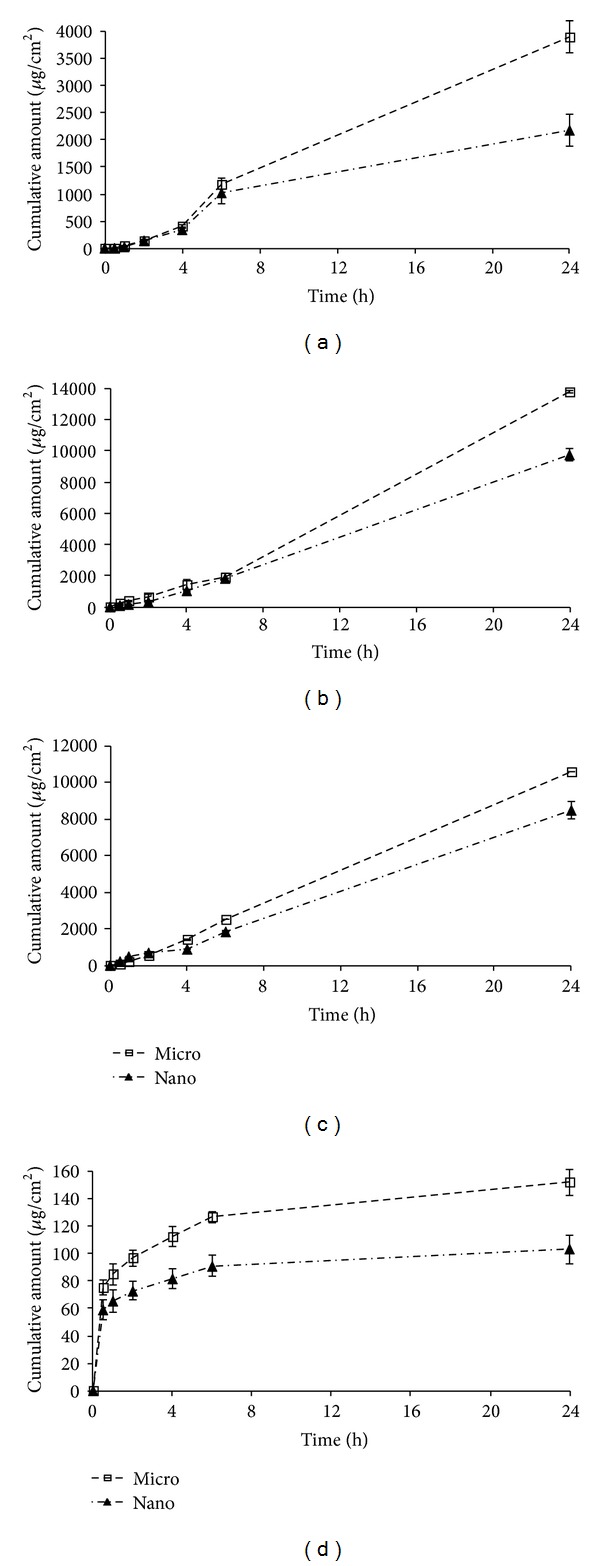
*In vitro* penetration-time profile of micronized/nanonized alaptide through skin from buffer (a) and from the following pharmaceutical compositions: gel (b), cream (c), and ointment (d). Points with error bars represent mean values of cumulative permeations (mean ± SD) determined in five experiments.

Cumulative permeated amounts per unit area were calculated from individual experimental data; see Table 1. The dependences of the cumulative amounts per unit area of the micronized/nanonized alaptide permeated through the porcine ear skin on the time [h] (the permeation profiles of micronized and nanonized forms of alaptide from various media) are illustrated in Figures 2(a)–2(d).

The general results of alaptide alone (only from buffer without any formulation) showed that micronized alaptide permeated significantly more than nanonized alaptide; nevertheless, permeation of micronized alaptide was really low. The amount of nanonized alaptide that permeated through the skin was approximately 2-fold less than the amount of permeated micronized alaptide. When alaptide was formulated in semisolid formulations, the observed *in vitro* transdermal permeation was higher for gel and cream than for buffer. In general it can be stated that more hydrophilic semisolid formulation means higher permeation, which is connected with the moisturizing effect on *stratum corneum* [29] and the presence of propylene glycol compared with the ointment composition. The permeation of both forms of alaptide through the skin from ointment was minimal; nevertheless, more alaptide permeated from ointment within 1 h than from buffer. In fact, it is well known that viscous formulations reduce the diffusion coefficient of the molecule in the vehicle, thus retarding or avoiding its skin partitioning, and hence absorption. In addition, extremely lipophilic formulations compete with *stratum corneum* lipophilicity, hampering the *stratum corneum* partitioning of the penetrating agent. In contrast, occlusive formulations may favor a moderate increase in absorption, as previously mentioned [21, 29, 39].

The field of nanoparticle drug delivery to the skin has progressed over the last dozen years to a point where there are well-characterized tools that have the capacity for customized pharmacokinetics. Although in general transport mechanisms support nanomaterial penetration [40–43], the promise of nanoparticle-mediated drug delivery into the epidermis and dermis without barrier modification has met with little success. It can be stated that the penetration of particulate materials into the skin is a very complex issue. Therefore, the interaction of nanoparticles with the skin and especially skin models is an intriguing field. However, the data obtained do not show a clear image of the effect of nanocarriers. In particular the penetration of such particles is an open and controversially discussed issue. The literature reports different results mainly on pig or murine skin showing strong penetration (pig and mouse) or the opposite. Looking only at the sizes of the particles, no conclusive picture can be obtained either. The fact that nanoparticles do not penetrate/permeate or they expressed decreased penetration/permeation was described and discussed in some recent papers [20–24, 44].

**Figure 3:**
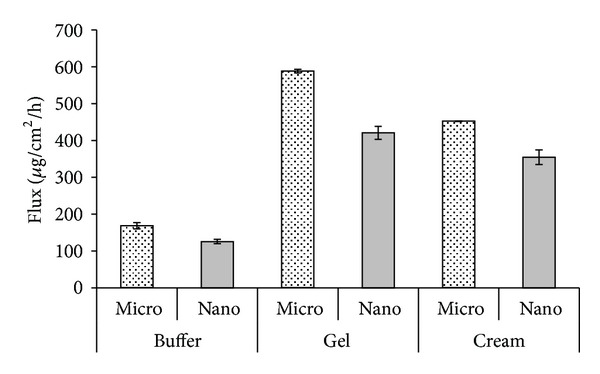
Range of nonsteady permeation flux (mean of five experiments ± SD) of micronized and nanonized alaptide from various media.

Due to the small number of time intervals in which permeation of alaptide was determined as well as the fact that in the time interval 6–12 h samples were not drawn, it is not possible to evaluate steady-state permeation fluxes. Therefore, apparent fluxes, the corresponding (*T*_lag_), and permeability coefficients (*K*_*p*_) were calculated from linearized dependences of six determined cumulative permeated amounts per unit area on time for buffer, gel, and cream formulations (they showed correlation coefficients (*r*) ranging from 0.980 to 0.999). Only the apparent flux of nanoparticles of alaptide in buffer was evaluated from the interval 0.5–4 h. The following permeation parameters are presented in Table 2: nonsteady-state flux (*J*), corresponding lag time (*T*_lag_), and apparent permeability coefficient (*K*_*p*_). Although these values are only informative, they can serve for comparison of permeation effectiveness of alaptide from buffer and hydrophilic semisolid formulations. The values of nonsteady-state permeation fluxes are illustrated in Figure 3.

It was not possible to calculate the above mentioned parameters for ointment composition with both forms of alaptide. In this case, cumulative permeated amounts of alaptide per unit area were more than one order lower compared with other investigated systems, and it can be stated that ointment medium, due to its constitution and small content of water, did not support/inhibited permeation of alaptide from the composition. Thus, alaptide with minimal permeation through the skin without achievement of systemic concentration can curatively act only in the upper skin layer, which is desirable from the point of view of various skin defects. Nevertheless also in case of this medium the obvious trend discussed above is evident, namely, the fact that the permeation of microparticles was higher than that of nanoparticles.

The permeability of alaptide from the evaluated media was significantly influenced by the composition (see Figure 3), especially by hydrophilicity of the whole formulation and proportions of possible surfactant/cosurfactant in the mixture.

The apparent permeability coefficient *K*_*p*_ representing absorption rate showed a wide range from 58.80 × 10^−3^ cm/h (micro gel) to 12.54 × 10^−3^ cm/h (nano buffer), that is, log⁡⁡*K*_*p*_ ranging from −1.23 to −1.90. It is evident again that nanonized alaptide from all the media showed lower permeation, and the penetration effectiveness of alaptide is influenced by formulation. According to the values of the logarithm of the permeability coefficient, it is possible to state that the permeation of micronized alaptide from buffer is close to nicotine (log⁡⁡*K*_*p*_ = −1.77) [45], while permeation of the nanonized alaptide from buffer is similar to sufentanil (log⁡⁡*K*_*p*_ = −1.92) [45]. It can be stated that penetration ability of alaptide is not small. Permeation through the skin from buffer of both forms of alaptide met quite good correlation with the result of regression analysis (4) of QSAR approach for skin permeability that was published by Barratt [45]:

(4) $$\begin{array}{c} \log P_{c}=-0.00734\text{MV}+0.769\log P-2.7713, \end{array}$$

where *P*_*c*_ is permeability coefficient, MV is molecular volume (predicted by CS ChemOffice Ultra ver. 10.0 (CambridgeSoft, Cambridge, MA, USA) expressed as a molar refractivity MR), and log⁡⁡*P* is logarithm of octanol-water partition coefficient *P*_o/w_ (for alaptide MR = 47.27 cm^3^/mol and experimental log⁡⁡*P*_o/w_ = 1.39 [8, 9]). The calculated value log⁡⁡*P*_*c*_ = −2.05 quite corresponds to experimentally found logarithm values of the permeability coefficient of micronized (log⁡⁡*K*_*p*_ = −1.77) and nanonized (log⁡⁡*K*_*p*_ = −1.90) alaptide from buffer, that is, without any modifier of permeation. Consequently, it can be stated that apparent permeability coefficients calculated from linearized dependences of cumulative permeated amounts on time (0.5–6 h for nanobuffer and 0.5–24 h for other investigated systems) can be used for comparison of permeation of both forms of alaptide from various media/systems.

Based on the above mentioned facts, it can be assumed that the evaluated pharmaceutical compositions can be used with success in combination with anti-inflammatory drugs, analgesics, antimicrobial chemotherapeutics, and/or antihemorrhagics. As was mentioned in the introduction, alaptide administered topically/locally supports/induces much faster regeneration and healing of the skin than in the case when only drugs alone from the above mentioned therapeutic groups are present in the formulation. The selection of the micronized or nanonized form of alaptide and the type of formulation can influence the depth and the rate of permeation to the skin, that is, the curative effect. Such pharmaceutical compositions containing alaptide can be used for formulation of alaptide alone or for combination of alaptide with other drugs and can be applied for treatment of skin and mucosal lesions, for example, burns, raws, decubitus, and venous ulcerations. The therapy of such skin lesions is very difficult, long-standing, and not always quite successful; therefore, it is suitable to have available as broad variety of effective therapeutics as possible [9].

## 4. Conclusion

In general, it can be concluded that nanonized alaptide permeated through PAMPA more than the micronized form. Different observations were obtained for permeation through the skin, where micronized alaptide permeated ca. 2-fold more from the buffer and other semisolid pharmaceutical compositions than the nanonized form; nevertheless, hydrophilic and hydrophobic media significantly influenced the permeation of alaptide. Both forms of alaptide permeated more effectively from hydrophilic gel and cream, where also the transdermal enhancer propylene glycol was present, than from buffer. The permeation of both forms of alaptide through the skin from ointment was minimal. The constitution of ointment did not support permeation of alaptide; thus, alaptide with minimal permeation through the skin without achievement of systemic effect can act only in the upper skin layer, which is desirable from the point of view of various skin defects.

## Figures and Tables

**Table 1:** Cumulative permeated amounts *Q*_*t*_ per unit area (*μ*g/cm^2^) of micronized/nanonized alaptide from buffer and from various semisolid formulations achieved in *in vitro *transdermal permeation experiments performed using Franz diffusion cell. Cumulative permeations are expressed as mean ± SD (*n* = 5 experiments).

Time (h)	Alaptide	Cumulative permeated amounts *Q*_*t*_ (*μ*g/cm^2^)			
		Buffer	Gel	Cream	Ointment
0.5	Micro	12.6 ± 1.9^a^	220.5 ± 11.1^abc^	89.9 ± 4.0^a^	75.3 ± 5.4^cd^
	Nano	6.9 ± 2.0^a^	105.7 ± 7.3^a^	246.7 ± 21.5^a^	58.9 ± 7.4^a^
1	Micro	40.1 ± 8.0^a^	401.6 ± 30.9^c^	206.7 ± 8.9^a^	84.8 ± 7.3^de^
	Nano	18.5 ± 7.6^a^	165.6 ± 7.0^ab^	488.1 ± 14.2^b^	65.0 ± 8.2^ab^
2	Micro	150.8 ± 7.2^a^	626.4 ± 211.1^d^	568.7 ± 7.2^b^	96.7 ± 5.9^fg^
	Nano	147.3 ± 9.2^a^	342.7 ± 79.9^bc^	676.07 ± 21.9^b^	72.9 ± 6.7^bc^
4	Micro	418.3 ± 24.6^b^	1474.5 ± 306.9^f^	1414.5 ± 21.3^d^	112.4 ± 7.3^h^
	Nano	345.9 ± 21.8^b^	1027.5 ± 28.0^e^	917.5 ± 13.5^c^	81.2 ± 7.2^cd^
6	Micro	1189.0 ± 105.9^d^	1927.6 ± 241.2^g^	2533.7 ± 9.2^f^	126.4 ± 3.7^i^
	Nano	1029.3 ± 212.6^c^	1850.9 ± 27.7^g^	1863.8 ± 24.1^e^	90.9 ± 7.6^ef^
24	Micro	3897.1 ± 295.6^f^	13766.8 ± 92.8^i^	10602.6 ± 18.2^h^	151.9 ± 9.5^j^
	Nano	2178.0 ± 295.7^e^	9774.1 ± 390.3^h^	8499.4 ± 461.0^g^	103.0 ± 10.3^gh^

The means followed by different letters are significantly different at *P* = 0.05. The analysis was performed for both micro- and nanoforms of alaptide within individual studied system (buffer as well as 3 semisolid formulations).

**Table 2:** Selective permeation parameters of micronized and nanonized alaptide through full-thickness pig ear skin from various media: nonsteady-state permeation flux (*J*), corresponding lag time (*T*_lag_), and apparent permeability coefficient (*K*_*p*_).

Medium	Alaptide	*J* (*μ*g/cm^2^/h)	*T*_lag_ (h)	*K*_*p*_ × 10^−3 ^ (cm/h)
Buffer	Micro	168.4 ± 8.7	0.69 ± 0.38	16.84
	Nano	125.4 ± 6.0*	0.76 ± 0.01*	12.54
Gel	Micro	588.0 ± 4.8	1.03 ± 0.23	58.80
	Nano	420.6 ± 17.5	0.99 ± 0.09	42.06
Cream	Micro	452.2 ± 0.8	0.57 ± 0.01	45.22
	Nano	354.4 ± 19.7	0.27 ± 0.03	35.44

*0.5–4 h.

## References

[1] De Souza Russo EM, Russo VFT

[2] Hashimoto K, Nakata K, Sakanaka M, Tanaka J

[3] Choi SG, Baek EJ, Davaa E (2013). Topical treatment of the buccal mucosa and wounded skin in rats with a triamcinolone acetonide-loaded hydrogel prepared using an electron beam. *International Journal of Pharmaceutics*.

[4] Kasafirek E, Sturc A, Roubalova A (1992). Linear tri- and tetrapeptides acting as prodrugs. *Collection of Czechoslovak Chemical Communications*.

[5] Kasafirek E, Rybak M, Krejci I, Sturc A, Krepela E, Sedo A (1992). Two-step generation of spirocyclic dipeptides from linear peptide ethyl ester precursors. *Life Sciences*.

[6] Celis ME, Taleisnik S, Walter R (1971). Regulation of formation and proposed structure of the factor inhibiting the release of melanocyte-stimulating hormone. *Proceedings of the National Academy of Sciences of the United States of America*.

[7] Petersson M, Uvnäs-Moberg K (2004). Prolyl-leucyl-glycinamide shares some effects with oxytocin but decreases oxytocin levels. *Physiology and Behavior*.

[8] Radl S, Kasafirek E, Krejci I (1990). Alaptide. *Drugs of the Future*.

[9] Jampílek J, Opatrilova R, Rezacova A, Oktabec Z, Dohnal J

[10] Douša M, Lemr K (2011). Liquid chromatographic method for enantiopurity control of alaptide using polysaccharide stationary phases. *Journal of Separation Science*.

[11] Maixner J, Rohlíček J, Kratochvíl B, Šturc A (2009). X-ray powder diffraction data for alaptide, 8(S)-methyl-6,9-diazaspiro/4,5/decane-7,10-dione or cyclo(l-alanyl-1-amino-1-cyclopentan carbonyl), cyclo(l-Ala-Acp). *Powder Diffraction*.

[12] Julinek O, Setnicka V, Rezacova A, Dohnal J, Vosatka V, Urbanova M (2010). Product of alaptide synthesis: determination of the absolute configuration. *Journal of Pharmaceutical and Biomedical Analysis*.

[13] Burns T, Breathnach S, Cox N, Griffiths C (2004). *Rook's Textbook of Dermatology*.

[14] James W, Berger T, Elston D (2006). *Andrews' Diseases of the Skin: Clinical Dermatology*.

[15] Watt FM (1988). The epidermal keratinocyte. *BioEssays*.

[16] Vanzura J, Kosar K, Kasafirek E (1986). Inhibition of proliferative activity by cyclic dipeptides: spirocyclic derivatives of 1-aminocyclopentanecarboxylic acid. *Toxicology Letters*.

[17] Lapka R (1991). Pharmacokinetics and brain entry of alaptide, a novel nootropic agent, in mice, rats and rabbits. *Journal of Pharmacy and Pharmacology*.

[18] Kosar K, Vanzura J (1988). Embryotoxicity of L-prolyl-L-leucyl-glycinamide, cyclo(1-amino-cyclopentanecarbonyl-alanyl) and cyclo(glycyl-leucyl), new potential neuropeptides in chick embryos. *Pharmazie*.

[19] http://www.bioveta.cz/en/veterinary-division/products/new-products-for-dogs-and-cats/alaptid-veterinary-ointment.html.

[20] Schneider M, Stracke F, Hansen S, Schaefer UF (2009). Nanoparticles and their interactions with the dermal barrier. *Dermatoendocrinol*.

[21] Baroli B (2010). Penetration of nanoparticles and nanomaterials in the skin: fiction or reality?. *Journal of Pharmaceutical Sciences*.

[22] Prow TW, Grice JE, Lin LL (2011). Nanoparticles and microparticles for skin drug delivery. *Advanced Drug Delivery Reviews*.

[23] Campbell CSJ, Contreras-Rojas LR, Delgado-Charro MB, Guy RH (2012). Objective assessment of nanoparticle disposition in mammalian skin after topical exposure. *Journal of Controlled Release*.

[24] Kimura E, Kawano Y, Todo H, Ikarashi Y, Sugibayashi K (2012). Measurement of skin permeation/penetration of nanoparticles for their safety evaluation. *Biological & Pharmaceutical Bulletin*.

[25] Kansy M, Senner F, Gubernator K (1998). Physicochemical high throughput screening: parallel artificial membrane permeation assay in the description of passive absorption processes. *Journal of Medicinal Chemistry*.

[26] Avdeef A, Tsinman O (2006). PAMPA—a drug absorption in vitro model: 13. Chemical selectivity due to membrane hydrogen bonding: in combo comparisons of HDM-, DOPC-, and DS-PAMPA models. *European Journal of Pharmaceutical Sciences*.

[27] Tam KY, Velicky M, Dryfe RAW, Mandic Z (2012). The importance of and different approaches to permeability determination. *Physico-Chemical Methods in Drug Discovery and Development*.

[28] Franz TJ (1975). Percutaneous absorption. On the relevance of in vitro data. *Journal of Investigative Dermatology*.

[29] Jampilek J, Brychtova K (2012). Azone analogues: classification, design, and transdermal penetration principles. *Medical Research Review*.

[30] Jampilek J (2013). Transdermal application of drugs and techniques affecting skin barrier. *Journal of Bioequivalence & Bioavailability*.

[31] Jacobi U, Kaiser M, Toll R (2007). Porcine ear skin: an in vitro model for human skin. *Skin Research and Technology*.

[32] Herkenne C, Naik A, Kalia YN, Hadgraft J, Guy RH (2006). Pig ear skin ex vivo as a model for in vivo dermatopharmacokinetic studies in man. *Pharmaceutical Research*.

[33] Meyer W, Schwarz R, Neurand K (1978). The skin of domestic mammals as a model for the human skin, with special reference to the domestic pig. *Current Problems in Dermatology*.

[34] Wu H, Ramachandran C, Weiner ND, Roessler BJ (2001). Topical transport of hydrophilic compounds using water-in-oil nanoemulsions. *International Journal of Pharmaceutics*.

[35] Akhtar N, Rehman MU, Khan HMS, Rasool F, Saeed T, Murtaza G (2011). Penetration enhancing effect of polysorbate 20 and 80 on the in vitro percutaneous absorption of L-ascorbic acid. *Tropical Journal of Pharmaceutical Research*.

[36] Panigrahi L, Pattnaik S, Ghosal SK (2005). The effect of pH and organic ester penetration enhancers on skin permeation kinetics of terbutaline sulfate from pseudolatex-type transdermal delivery systems through mouse and human cadaver skins. *AAPS PharmSciTech*.

[37] Huang CT, Tsai MJ, Lin YH (2013). Effect of microemulsions on transdermal delivery of citalopram: optimization studies using mixture design and response surface methodology. *International Journal of Nanomedicine*.

[38] Rhee Y-S, Choi J-G, Park E-S, Chi S-C (2001). Transdermal delivery of ketoprofen using microemulsions. *International Journal of Pharmaceutics*.

[39] Kennish L, Reidenberg B (2005). A review of the effect of occlusive dressings on lamellar bodies in the stratum corneum and relevance to transdermal absorption. *Dermatology Online Journal*.

[40] Bhushan B (2004). *Handbook of Nanotechnology*.

[41] Buzea C, Pacheco II, Robbie K (2007). Nanomaterials and nanoparticles: sources and toxicity. *Biointerphases*.

[42] Corredor E, Testillano PS, Coronado M-J (2009). Nanoparticle penetration and transport in living pumpkin plants: in situ subcellular identification. *BMC Plant Biology*.

[43] Verma A, Stellacci F (2010). Effect of surface properties on nanoparticle-cell interactions. *Small*.

[44] Watkinson AC, Bunge AL, Hadgraft J, Lane ME (2013). Nanoparticles do not penetrate human skin-a theoretical perspective. *Pharmaceutical Research*.

[45] Barratt MD (1995). Quantitative structure-activity relationships for skin permeability. *Toxicology in Vitro*.

